# Supplementary material for: CslA and GlxA from Streptomyces lividans form a functional cellulose synthase complex
Source: Appl Environ Microbiol. 2024 Apr 1;90(4):e02087-23. doi: 10.1128/aem.02087-23 (PMC11022532; doi:10.1128/aem.02087-23)
Supplement: Supplemental material — Figures S1 to S7; Tables S1 to S3. [file aem.02087-23-s0001.docx]

Supporting Information

**CslA and GlxA from *Streptomyces lividans* form a functional cellulose synthase complex**

Xiaobo Zhong, Simone Nicolardi, Ruochen Ouyang, Manfred Wuhrer, Chao Du, Gilles van Wezel, Erik Vijgenboom, Ariane Briegel, Dennis Claessen

Correspondence to: [d.claessen@biology.leidenuniv.nl](mailto:d.claessen@biology.leidenuniv.nl)

This PDF files includes:

Supplementary Figures S1-S7

Supplementary Tables S1-S3


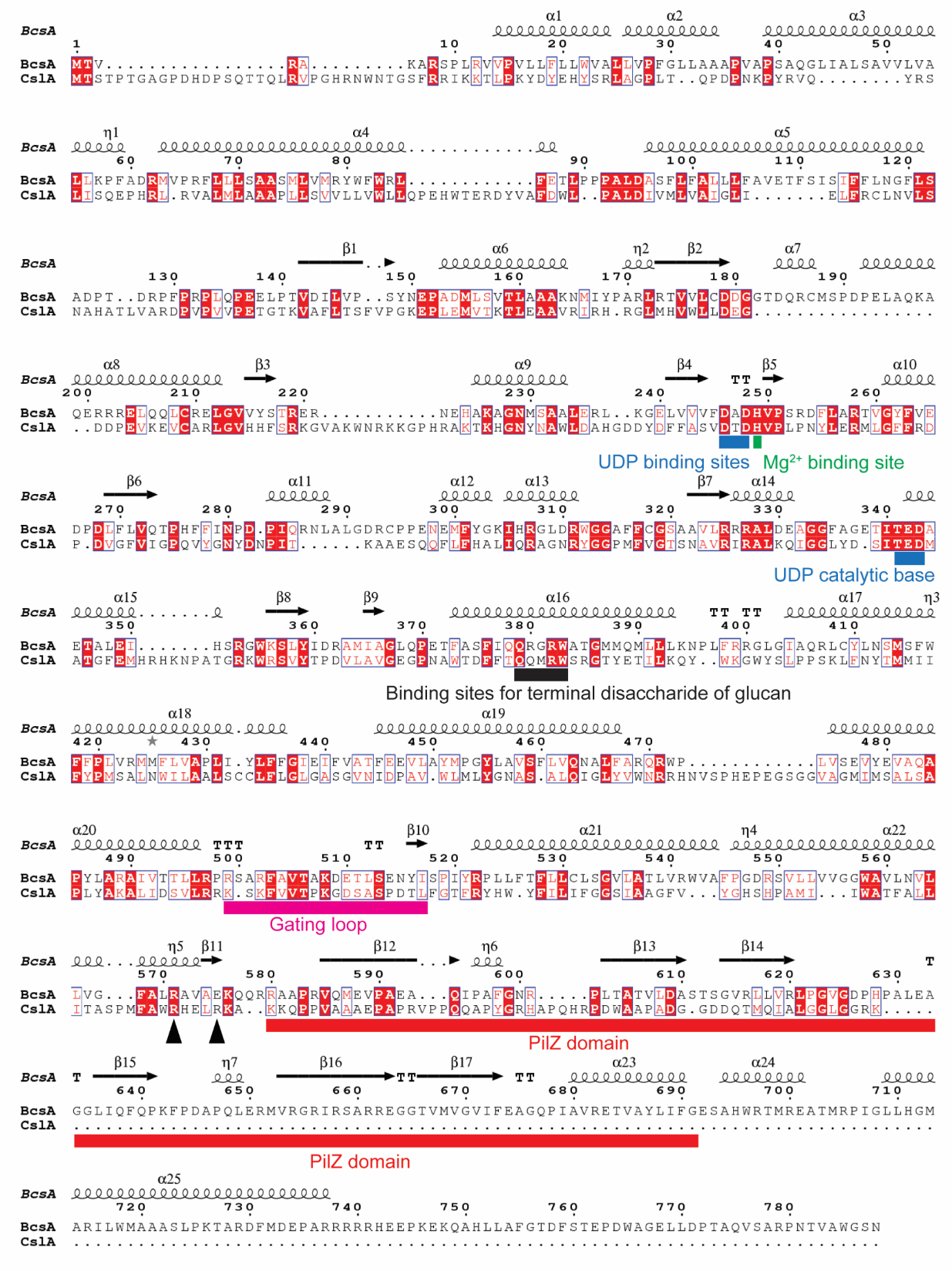


**Figure S1. Sequence alignment of CslA from *Streptomyces lividans* and BcsA from *Rhodobacter spaeroides***. Sequence alignment was performed by ESPript 3.0 and annotated using Adobe Illustrator software. The sequence alignment shows that CslA of *Streptomyces coelicolor* (UniProt entry: Q9RDB5) and BcsA of *Rhodobacter sphaeroides* (UniProt entry: A0A3G6W9S6) share 27% sequence identity. Signature motifs of CslA are indicated,including the UDP-coordinating bases (D, D, D), the Mg^2+^ binding site (H), binding sites for the terminal disaccharide of the glucan (Q(Q/R)XRW), the gating loop (FXVTXK), and elements of a putative partial PilZ domain (RXXXR, indicated by black arrows).


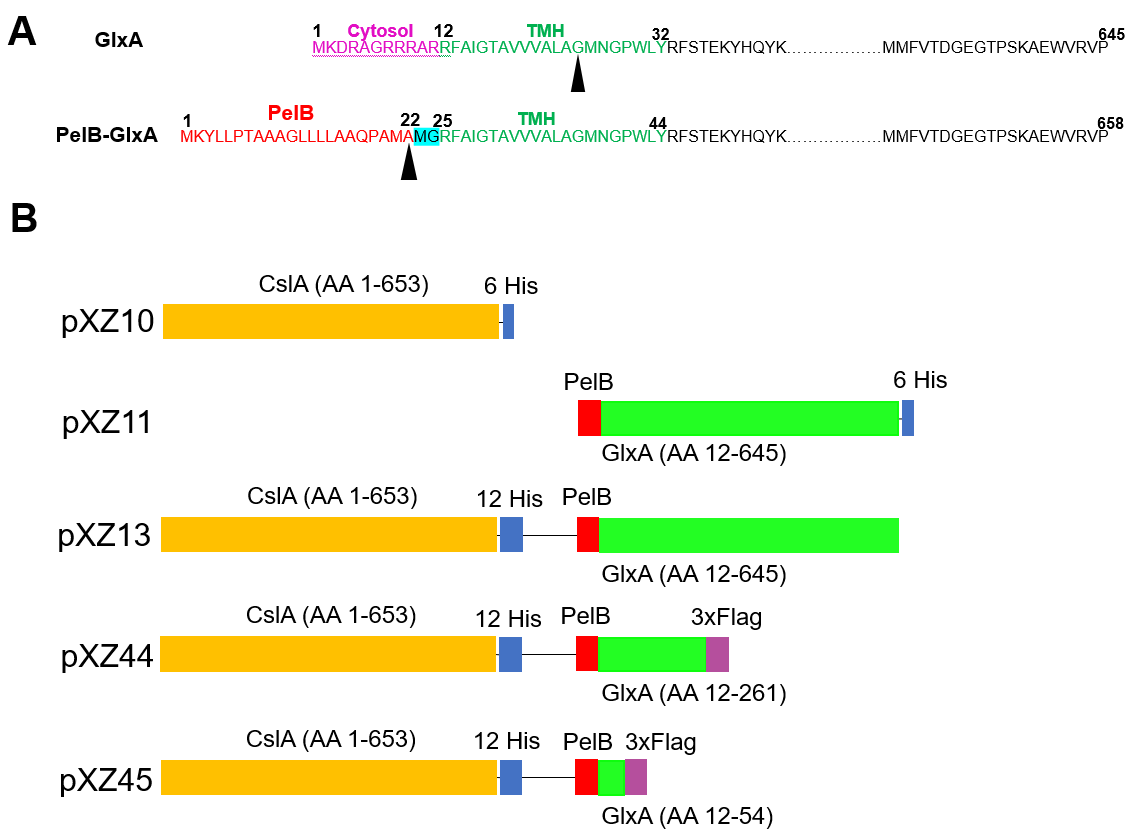


**Figure S2. Strategy for expression of the CslA-GlxA complex.** **(A)** Amino acid sequence of full length GlxA and the PelB-GlxA fusion. GlxA comprises a cytosolic part (AA 1-11), a transmembrane helix (TMH, AA 12-32) and an extracellular sequence (AA 33-645). In PelB-GlxA, the cytosolic sequence was replaced by a PelB leader peptide, in which PelB and GlxA are connected via a Met-Gly dipeptide (indicated in magenta) due to the *NcoI* restriction enzyme that was used. Arrows indicate the predicted peptidase cleavage sites. The transmembrane helices (TMH) were predicted using TMHMM-v2.0. **(B)** Inserts used to express CslA (in pXZ10), GlxA (in pXZ11), the CslA-GlxA complex (in pXZ13), the CslA-GlxA^12-261^ complex (in pZX44) and CslA-GlxA^12-54^ (in pXZ45).


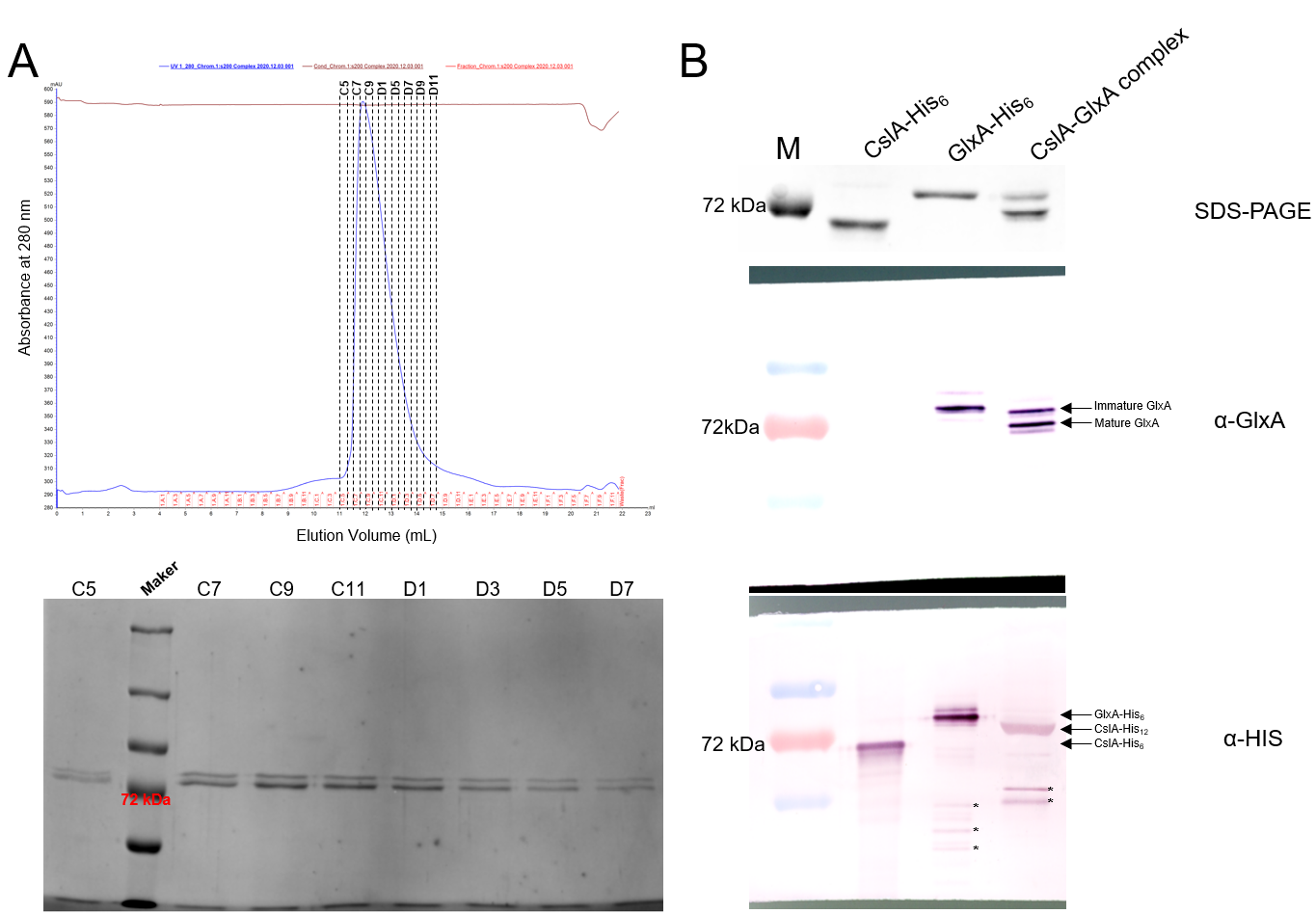


**Figure S3. Analysis of the CslA-GlxA complex. (A)** Size exclusion chromatography (SEC) analysis of the CslA-GlxA complex, which was done using a BioRad GNC^TM^ system equipped with a Superdex 200 Increase 10/300 GL column. 10 µl of each fraction was separated on a 7.5% SDS-PAGE gel, which was subsequently stained with Coomassie Brilliant Blue (bottom panel). **(B)** Immunoblotting analysis of CslA (CslA-His_6_), GlxA (GlxA-His_6_), and the the CslA-GlxA complex (CslA-His_12_-GlxA). Samples containing 5 µg protein were loaded and separated on 7.5% SDS-PAGE gels and subsequently stained with Coomassie Brilliant Blue (top panel) or analysed using immunoblotting with antibodies directed against GlxA (middle panel) or the His-tags (bottom panel). In the CslA-His_12_-GlxA complex, two GlxA bands are visible corresponding to immature GlxA (without the Tyr-Cys cofactor, upper band) and mature GlxA (with the Tyr-Cys cofactor, lower band). Please note that CslA has a larger molecular weight in the complex due to the His_12_-tag. ***** Asterisks mark non-specific signals or potential degradation products.


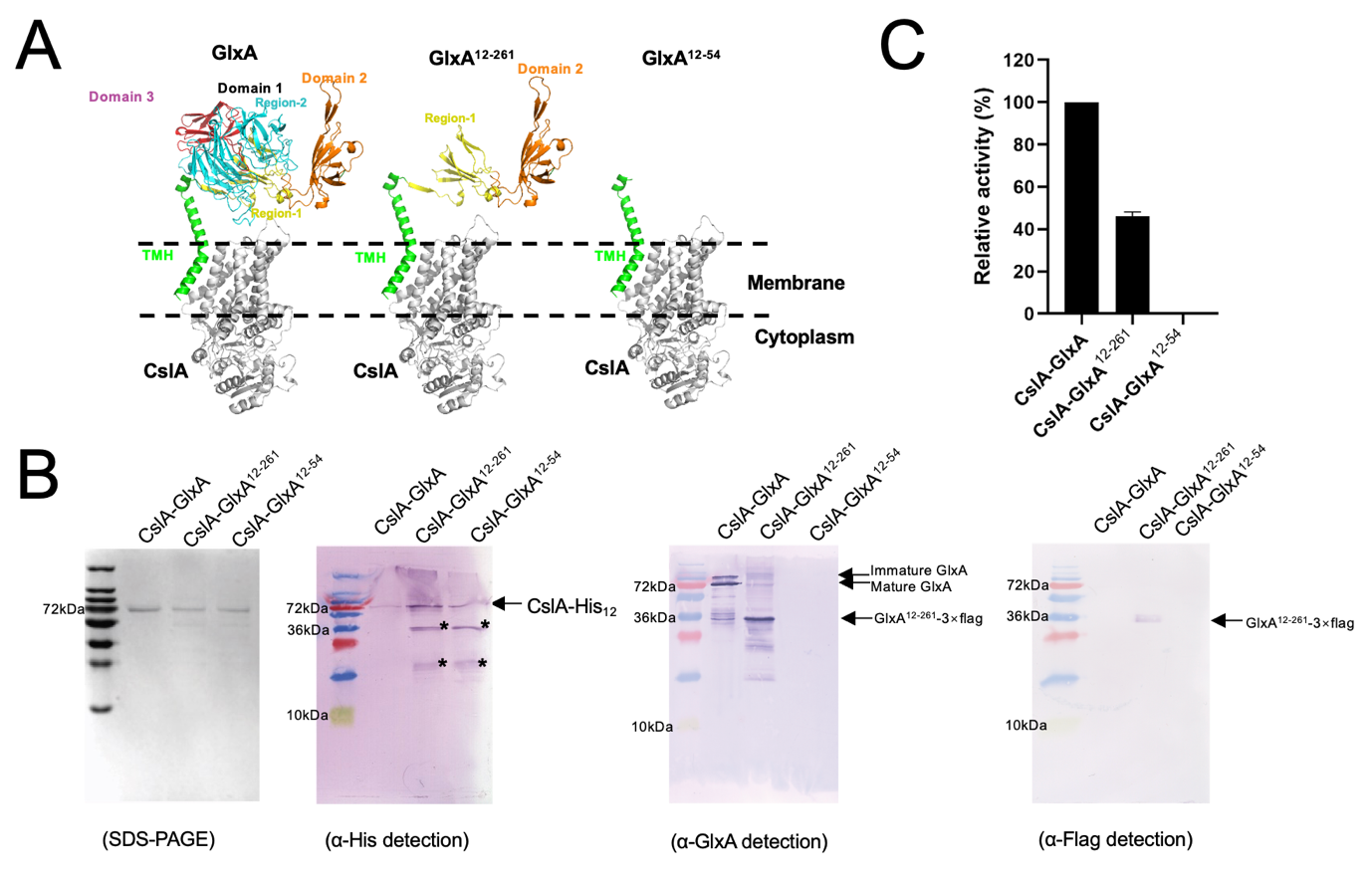


**Figure S4. The TMH region of GlxA is not sufficient for formation of the GlxA-CslA complex. (A)** Schematic overview of GlxA truncations (GlxA^12-261^ and GlxA^12-54^) used in pull-down assays with CslA. **(B)** Immunoblotting analyses of protein complexes obtained using pull-down assays. For each well, 2 µg protein was loaded. ***** Asterisks mark non-specific signals or potential degradation products. Please note that GlxA^12-54^ is not sufficient to form a complex with CslA. **(C)** Activity analyses of purified complexes. Reactions were performed by incubating 0.05 mg mL^-1^ of each protein complex with 5 mM UDP-Glc, 5 mM cellobiose, 20 mM MgCl_2_ and 30 μM cyclic-di-GMP at 37°C for 60 min. The catalytic activity of enzymes was quantified by measuring free UDP with the UDP-Glo^TM^ glycosyltransferase assay. The catalytic activity impacted by GlxA truncations was assessed relative to the ClsA-GlxA complex. Every measurement was performed in triplicate. Error bars represent the standard error of the mean.


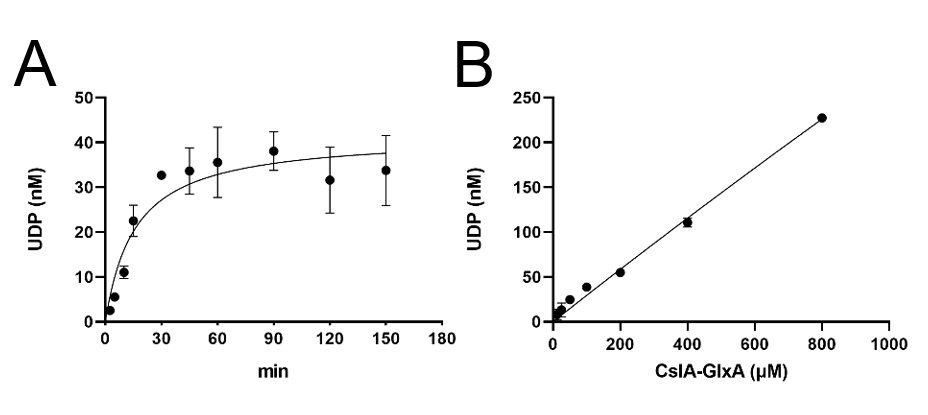


**Figure S5. Kinetics of cellulose synthesis by the purified CslA-GlxA complex. (A)** Time course of cellulose synthesis *in vitro* with UDP-Glc as the substrate. The data were fitted using Michaelis-Menten kinetics. A linear relationship is found between UDP release and protein concentration (0-1 mM) at 5 mM UDP-Glc **(B)**. Catalytic activity of CslA-GlxA was determined by quantifying the amount of released UDP. All reactions were conducted in triplicate. Error bars represent the standard error of the mean.


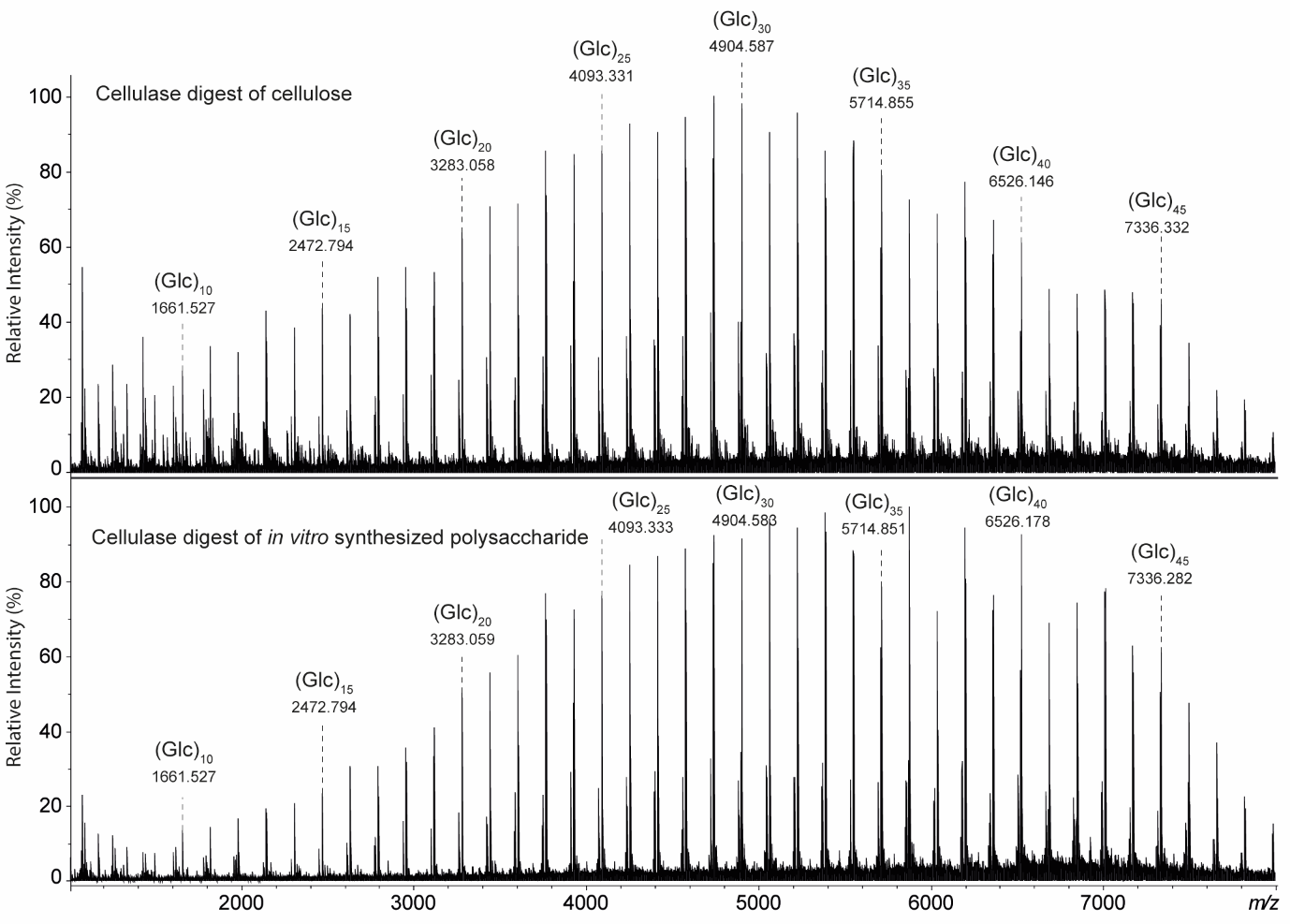


**Figure S6. MALDI-CID FT-ICR MS analysis of oligosaccharides from cellulase digests of cellulose and the *in vitro* synthesized glucan.** Enlargement of MALDI FT-ICR mass spectra in the *m/z*-range 1011-8000 from the analysis of the cellulase digest of cellulose (top) or the *in vitro* synthesized glucan (bottom).


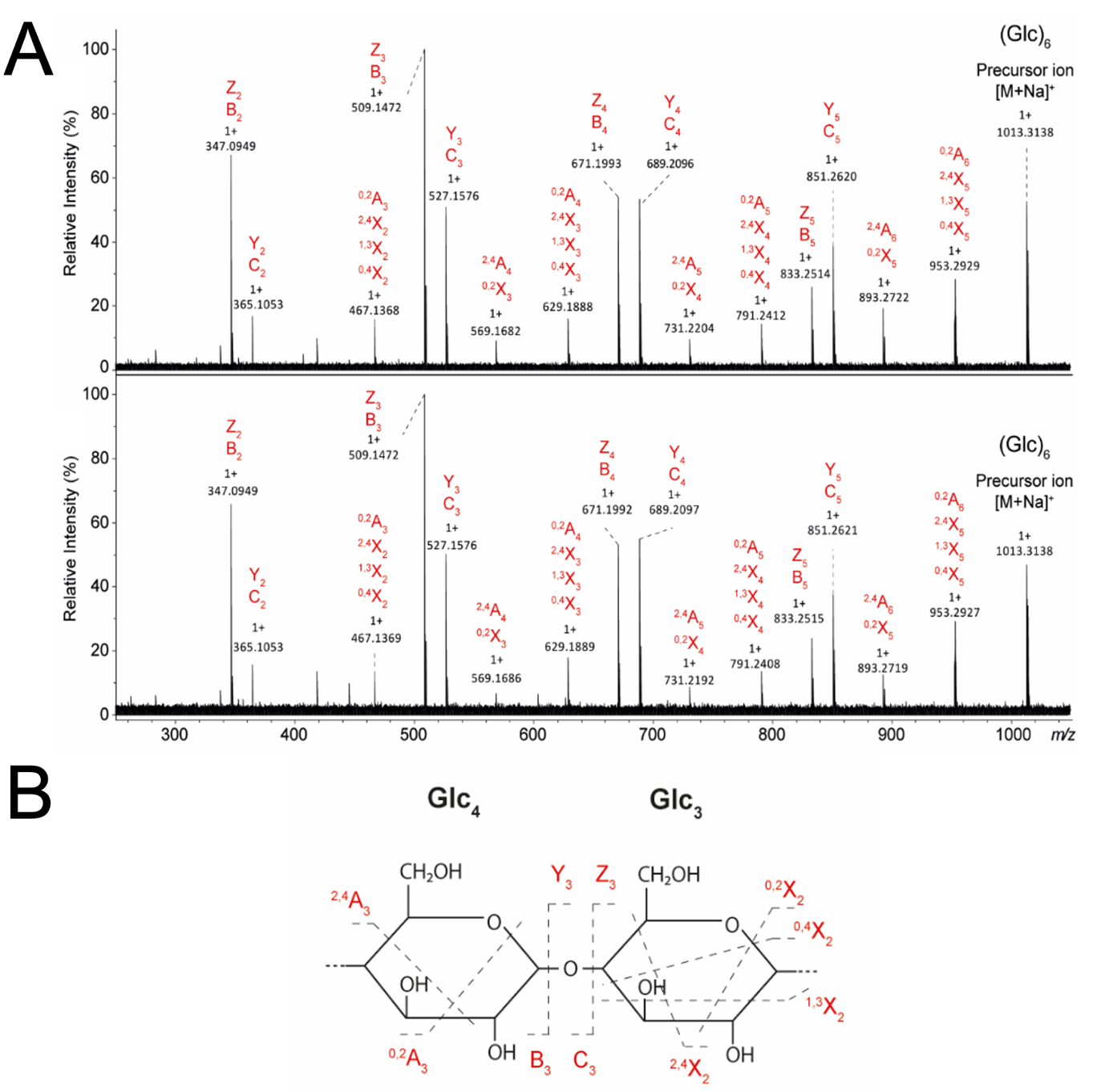


**Figure S7. Linkage analysis of hexaglucose fractions from the cellulase digests of cellulose and the in vitro synthesized glucan.** (**A**) Hexaglucose oligosaccharides obtained from Figure S7 were subjected to linkage analysis by MALDI-CID FT-ICR MS, in which top and bottom panels represent mass spectra of the analysis of the cellulase digest of cellulose and the in vitro synthesized glucan, respectively. Isomeric fragments are vertically aligned. The fragmentation scheme used for the assignments is indicated in (**B**).

**Table S1. Primers used in this study**

| **Name** **Sequence** | |
| --- | --- |
| 3187-F | CATGCATATGATGACGTCGACGCCGACGGGCG |
| 3187-R | CCCAAGCTTTTCCTTACGTCCCCCAAGTCCACCAAGGG |
| 3188-F | CATGCCATGGGACGCTTCGCGATAGGTACGGCG |
| 3188-R | CCCAAGCTTCGGCACCCGCACCCACTC |
| blaM-F | CCCAAGCTTGCGGTTCCACATGCTATAAGTTCCAGCCCATTACGCcacccagaaacgctggtgaaa |
| blaM_FL_-F | GGAATTCCATATGATGAGTATTCAACATTTCCGTGTCG |
| blaM-R | CCGGAATTCTTACCAATGCTTAATCAGTGAGGC |
| glxA_1-96_-F | GGAATTCCATATGAAAGACCGTGCCGGCCGC |
| glxA_1-96_-R | CCCAAGCTTGTAGAGCCACGGCCCGTTCATCC |
| glxA_Blam_-F | GGAATTCCATATGATGAAAGACCGTGCCGGCCGCCGC |
| glxA_Blam_-R | CCCAAGCTTCGGCACCCGCACCCACTCCGCCTTAC |
| pglxA-F | GGGAATTGTGAGCGGATAACAATTCCCC |
| pglxA-R | CCTTAATTAACTA CGGCACCCGCACCCACTC |
| cslA-F | GGGAATTGTGAGCGGATAACAATTCCCC |
| cslA-R | CCGGAATTCTCAGTGGTGGTGGTGGTGGTGGTGGTGGTGGTGGTGGTGCTCGAGTGC |
| Flag-F | gattataaggatcatgatggtgattataaggatcatgatatcgactacaaagacgatgacgacaagTAGTGCTGCCACCGCTGAGCAATAAC |
| GlxA261-R | GAAGTCCTTCTTGTCGAGGGCGAGC |
| GlxA54-R | GCCGTTGGCGGCCTTGTACTC |

**Table S2. Plasmids used in this study**

| **Name** | **Description** | **References** |
| --- | --- | --- |
| pXZ2 | pSET152 plasmid containing the *gapAp_cslZ* fragment. | (17) |
| pHJL401 | *E. coli/Streptomyces* shuttle vector containing the *blaM* gene | (33) |
| pXZ10 | pET21a plasmid used for expressing CslA-His_6_ | This work |
| pXZ11 | pET22b plasmid used for expressing PelB-GlxA-His_6_ | This work |
| pXZ12 | pETDueT-1 plasmid used for expressing PelB-GlxA in MCSII | This work |
| pXZ13 | pETDueT-1 plasmid used for expressing CslA-His_12_ and PelB-GlxA in MCS I and II, respectively | This work |
| pXZ36 | pSET152 plasmid expressing *blaM*_FL_ from the constitutive *gapAp* promoter | This work |
| pXZ42 | pSET152 plasmid expressing *glxA*_1-96_-*blaM*_NS_ from the constitutive *gapAp* promoter | This work |
| pXZ43 | pSET152 plasmid expressing *glxA_FL_*-*blaM*_NS_ from the constitutive *gapAp* promoter | This work |
| pXZ44 | pETDueT-1 plasmid used for expressing PelB-GlxA^12-262^-3×Flag in MCSII and CslA-His_12_ in MCSI | This work |
| pXZ45 | pETDueT-1 plasmid used for expressing PelB-GlxA^12-54^-3×Flag in MCSII and CslA-His_12_ in MCSI | This work |

**Table S3. Proteins used for the construction of the phylogenetic tree of Figure 1.**

| **Abbreviation** | **Protein name** | **Organism** | **UniProt Entry** |
| --- | --- | --- | --- |
| SCO_2836 | Cellulose synthase-like protein | *Streptomyces coelicolor* (strain ATCC BAA-471 / A3(2) / M145) | Q9RDB5 |
| SVEN_5601 | Cellulose synthase-like protein | *Streptomyces venezuelae* (strain ATCC 10712) | F2R878 |
| SCAB_57291 | Cellulose synthase-like protein | *Streptomyces scabiei* (strain 87.22) | [C9Z2P5](https://www.uniprot.org/uniprotkb/C9Z2P5/entry) |
| SAV_5219 | Cellulose synthase-like protein | [*Streptomyces avermitilis* (strain ATCC 31267)](https://www.uniprot.org/taxonomy/227882) | Q82CW9 |
| SGR_4704 | Cellulose synthase-like protein | *Streptomyces griseus* (strain JCM 4626/NBRC 13350) | [B1VW22](https://www.uniprot.org/uniprotkb/B1VW22/entry) |
| SLI_3187 | Cellulose synthase-like protein | *Streptomyces lividans* 1326 | [A0A7U9DT44](https://www.uniprot.org/uniprotkb/A0A7U9DT44/entry) |
| SCLAV_1986 | Cellulose synthase-like protein | *Streptomyces clavuligerus* (strain ATCC 27064) | B5H368 |
| E_C_BcsA | Bacterial cellulose synthase A | *Escherichia coli (strain K12)* | P37653 |
| K_X_BcsA | Bacterial cellulose synthase A | *Komagataeibacter xylinus* | P19449 |
| PfBcsA | Bacterial cellulose synthase A | *Pseudomonas fluorescens* | P58931 |
| RsBcsA | Bacterial cellulose synthase A | *Rhodobacter sphaeroides* | [A0A3G6W9S6](https://www.uniprot.org/uniprotkb/A0A3G6W9S6/entry) |
| AtCesA1 | Cellulose synthase A1 | *Arabidopsis thaliana* | [O48946](https://www.uniprot.org/uniprotkb/O48946/entry) |
| AtCesA2 | Cellulose synthase A2 | *Arabidopsis thaliana* | O48947 |
| AtCesA3 | Cellulose synthase A3 | *Arabidopsis thaliana* | Q941L0 |
| AtCesA4 | Cellulose synthase A4 | *Arabidopsis thaliana* | Q84JA6 |
| AtCesA5 | Cellulose synthase A5 | *Arabidopsis thaliana* | Q8L778 |
| AtCesA6 | Cellulose synthase A6 | *Arabidopsis thaliana* | [Q94JQ6](https://www.uniprot.org/uniprotkb/Q94JQ6/entry) |
| AtCesA7 | Cellulose synthase A7 | *Arabidopsis thaliana* | Q9SWW6 |
| AtCesA8 | Cellulose synthase A8 | *Arabidopsis thaliana* | Q8LPK5 |
| AtCesA9 | Cellulose synthase A9 | *Arabidopsis thaliana* | Q9SJ22 |
| AtCesA10 | Cellulose synthase A10 | *Arabidopsis thaliana* | Q9SKJ5 |
| AtCslA2 | Cellulose synthase-like protein A2 | *Arabidopsis thaliana* | Q9FNI7 |
| AtCslA7 | Cellulose synthase-like protein A7 | *Arabidopsis thaliana* | Q9ZQN8 |
| AtCslA9 | Cellulose synthase-like protein A9 | *Arabidopsis thaliana* | Q9LZR3 |
| AtCslB1 | Cellulose synthase-like protein B1 | *Arabidopsis thaliana* | O80898 |
| AtCslB2 | Cellulose synthase-like protein B2 | *Arabidopsis thaliana* | O80899 |
| AtCslB3 | Cellulose synthase-like protein B3 | *Arabidopsis thaliana* | Q8RX83 |
| AtCslB5 | Cellulose synthase-like protein B5 | *Arabidopsis thaliana* | Q0WT40 |
| AtCslB6 | Cellulose synthase-like protein B6 | *Arabidopsis thaliana* | O23386 |
| AtCslC2 | Cellulose synthase-like protein C2 | *Arabidopsis thaliana* | Q9ZQB9 |
| AtCslC4 | Cellulose synthase-like protein C4 | *Arabidopsis thaliana* | Q9LJP4 |
| AtCslC5 | Cellulose synthase-like protein C5 | *Arabidopsis thaliana* | Q9SB75 |
| AtCslC6 | Cellulose synthase-like protein C6 | *Arabidopsis thaliana* | Q9SRT3 |
| AtCslC8 | Cellulose synthase-like protein C8 | *Arabidopsis thaliana* | Q9SJA2 |
| AtCslE1 | Cellulose synthase-like protein E1 | *Arabidopsis thaliana* | Q8VZK9 |
| AtCslG1 | Cellulose synthase-like protein G1 | *Arabidopsis thaliana* | Q570S7 |
| AtCslG2 | Cellulose synthase-like protein G2 | *Arabidopsis thaliana* | Q8VYR4 |
| OsCesA1 | Cellulose synthase A1 | *Oryza sativa* | A2Y0X2 |
| OsCesA2 | Cellulose synthase A2 | *Oryza sativa* | A2XN66 |
| OsCesA3 | Cellulose synthase A3 | *Oryza sativa* | Q69V23 |
| OsCesA4 | Cellulose synthase A4 | *Oryza sativa* | Q5JN63 |
| OsCesA5 | Cellulose synthase A5 | *Oryza sativa* | Q851L8 |
| OsCesA6 | Cellulose synthase A6 | *Oryza sativa* | Q6YVM4 |
| OsCesA7 | Cellulose synthase A7 | *Oryza sativa* | Q9AV71 |
| OsCesA8 | Cellulose synthase A8 | *Oryza sativa* | Q84ZN6 |
| OsCesA9 | Cellulose synthase A9 | *Oryza sativa* | A2Z1C8 |
| OsCesA11 | Cellulose synthase A11 | *Oryza sativa* | Q69XK5 |
| OsCslA1 | Cellulose synthase-like protein A1 | *Oryza sativa* | Q7PC76 |
| OsCslA2 | Cellulose synthase-like protein A2 | *Oryza sativa* | Q7PC67 |
| OsCslA3 | Cellulose synthase-like protein A3 | *Oryza sativa* | Q67X45 |
| OsCslA4 | Cellulose synthase-like protein A4 | *Oryza sativa* | Q8S7W0 |
| OsCslA5 | Cellulose synthase-like protein A5 | *Oryza sativa* | Q7PC73 |
| OsCslA6 | Cellulose synthase-like protein A6 | *Oryza sativa* | Q6Z2T9 |
| OsCslA7 | Cellulose synthase-like protein A7 | *Oryza sativa* | Q7XIF5 |
| OsCslA9 | Cellulose synthase-like protein A9 | *Oryza sativa* | Q67VS7 |
| OsCslA11 | Cellulose synthase-like protein A11 | *Oryza sativa* | Q6YWK8 |
| OsCslC2 | Cellulose synthase-like protein C2 | *Oryza sativa* | Q69L19 |
| OsCslC9 | Cellulose synthase-like protein C9 | *Oryza sativa* | Q6AU53 |
| OsCslD1 | Cellulose synthase-like protein D1 | *Oryza sativa* | Q8W3F9 |
| OsCslD2 | Cellulose synthase-like protein D2 | *Oryza sativa* | A2YU42 |
| OsCslD3 | Cellulose synthase-like protein D3 | *Oryza sativa* | Q7EZW6 |
| OsCslD4 | Cellulose synthase-like protein D4 | *Oryza sativa* | Q2QNS6 |
| OsCslD5 | Cellulose synthase-like protein D5 | *Oryza sativa* | Q5Z6E5 |
| OsCslE1 | Cellulose synthase-like protein E1 | *Oryza sativa* | Q651X7 |
| OsCslE2 | Cellulose synthase-like protein E2 | *Oryza sativa* | Q0DXZ1 |
| OsCslF2 | Cellulose synthase-like protein F2 | *Oryza sativa* | Q84S11 |
| OsCslF3 | Cellulose synthase-like protein F3 | *Oryza sativa* | A2YMH5 |
| OsCslF4 | Cellulose synthase-like protein F4 | *Oryza sativa* | Q6ZF86 |
| OsCslF6 | Cellulose synthase-like protein F6 | *Oryza sativa* | Q84UP7 |
| OsCslF7 | Cellulose synthase-like protein F7 | *Oryza sativa* | Q94GM9 |
| OsCslF9 | Cellulose synthase-like protein F9 | *Oryza sativa* | Q7XHV0 |
| OsCslH1 | Cellulose synthase-like protein H1 | *Oryza sativa* | Q339N5 |
| OsCslH2 | Cellulose synthase-like protein H2 | *Oryza sativa* | Q7PC71 |
| OsCslH3 | Cellulose synthase-like protein H3 | *Oryza sativa* | Q7XUU0 |
